# Impact of maternal western diet-induced obesity on offspring mortality and peripheral endocannabinoid system in mice

**DOI:** 10.1371/journal.pone.0205021

**Published:** 2018-10-01

**Authors:** Pedro A. Perez, Nicholas V. DiPatrizio

**Affiliations:** University of California Riverside, School of Medicine, Division of Biomedical Sciences, Riverside CA, United States of America; Hospital Infantil Universitario Nino Jesus, SPAIN

## Abstract

Over two-thirds of adults in the United States are obese or overweight, which is largely due to chronic overconsumption of diets high in fats and sugars (i.e., Western diet). Recent studies reveal that maternal obesity may predispose offspring to development of obesity and other metabolic diseases; however, the molecular underpinnings of these outcomes are largely unknown. The endocannabinoid system is an important signaling pathway that controls feeding behavior and energy homeostasis, and its activity becomes upregulated in the upper small intestinal epithelium of Western diet-induced obese mice, which drives overeating. In the current investigation, we examined the impact of chronic maternal consumption of Western diet on the expression and function of the endocannabinoid system in several peripheral organs important for food intake and energy homeostasis in offspring. Female C57BL/6Tac mice were fed a Western diet or low-fat/no-sucrose control chow for 10 weeks, then males were introduced for mating. Dams were maintained on their respective diets through weaning of pups, at which time pups were maintained on low-fat/no-sucrose chow for 10 weeks. Neonates born from dams fed Western diet, when compared to those born from mice fed control chow, unexpectedly displayed increases in mortality that occurred exclusively within six days following birth (greater than 50% mortality). Males comprised a larger fraction of surviving offspring from obese dams. Furthermore, surviving offspring displayed transient increases in body mass for first two days post weaning, and no marked changes in feeding patterns and endocannabinoid levels in upper small intestinal epithelium, pancreas, and plasma, or in expression of key endocannabinoid system genes in the upper small intestinal epithelium and pancreas at 10 weeks post-weaning. Collectively, these results suggest that maternal diet composition greatly influences survival of neonate C57BL/6Tac mice, and that surviving offspring from dams chronically fed a Western diet do not display marked changes in body mass, eating patterns, or expression and function of the endocannabinoid system in several peripheral organs important for feeding behavior and energy homeostasis.

## Introduction

Over 70% of adults in the United States are overweight or obese, and childhood and adolescent obesity rates have more than tripled from the 1970s [1–4]. Diet-induced obesity (DIO) is preventable and associated with overconsumption of foods high in fats and sugars [i.e. a Western diet (WD)], which greatly increases risk of developing type-2 diabetes and other metabolic diseases [5, 6]. Furthermore, there is increasing incidence of obesity in women during childbearing age and increases in gestational diabetes during pregnancy [7, 8]. Evidence also links gestational diabetes or obesity to increased risk of developing type-2 diabetes in, both, mothers and their offspring [9, 10]. Studies in rodent models of maternal DIO reveal changes in offspring taste preference, stress responses, adiposity, and weight gain; however, the molecular mechanisms underlying these behavioral and metabolic outcomes are poorly understood but may include dysregulation of the endocannabinoid (eCB) system [11–18]. Indeed, substantial evidence suggests that the eCB system serves critical roles in food intake and energy balance [19–22].

The eCB system is comprised of the lipid-derived signaling molecules, the eCBs, which include the well-characterized 2-arachidonoyl-*sn-*glycerol (2-AG) and anandamide (AEA), and their biosynthetic and degradative enzymes, and receptors [i.e., cannabinoid receptor subtype-1 and subtype-2 (CB_1_R and CB_2_R)] [23, 24]. The eCB system plays important roles in nearly all physiological functions associated with energy balance, including pancreatic endocrine function [21]. CB_1_R activity on β-cells in the pancreas impact their function by directly inhibiting insulin receptor signaling, as well as in utero pancreatic endocrine islet development [25–27]. Increasing evidence also suggests that CB_1_R activation within the islets of Langerhans stimulates insulin secretion through a cAMP- and calcium-dependent mechanism [28–31]; however, other groups report inhibitory actions on insulin secretion [32–34]. Nonetheless, changes in pancreatic eCB signaling during development may predispose offspring to perturbations in glucose homeostasis.

The eCB system in, both, the brain and periphery plays an important role in controlling feeding behaviors [19–21, 35–38]. CB_1_R antagonists are widely reported to inhibit palatable food intake in lean and DIO rodents, and improve a multitude of metabolic parameters [39–43], which highlights the therapeutic potential for cannabinoid receptor inhibitors to combat the growing obesity epidemic. In neonate mice, systemic administration of the CB_1_R antagonist/inverse agonist, SR141716, led to the failure of suckling and ultimately death, which underscores the importance of the eCB system in developing early feeding signals [44]. Furthermore, recent studies suggest a role for the eCB system in peripheral tissues–specifically the proximal small intestine–in the control of food intake [45, 46]. Indeed, 2-AG levels in the proximal small intestinal epithelium were elevated in, both, fasted lean male mice and non-fasted male mice maintained on WD for 60 days [40, 47, 48]. When compared to lean mice maintained on a low-fat/sugar control diet, mice fed WD rapidly gained body weight and were hyperphagic with increased daily caloric consumption and meal size [40]. Importantly, inhibiting peripheral CB_1_Rs with the peripherally-restricted neutral CB_1_R antagonist, AM6545, fully normalized eating patterns in mice fed WD for 60 days to those found in lean controls. These studies suggest that overeating associated with a WD is controlled by overactive eCB signaling at cannabinoid CB_1_Rs in the upper small intestinal epithelium. Thus, it is plausible that maternal DIO may impact eCB signaling in the proximal small intestine in offspring, with functional outcomes that may include dysregulation of food intake and energy balance.

This study aimed to identify the impact of a maternal WD on offspring body weight gain, feeding patterns, and expression and function of the eCB system in select peripheral organs important for food intake and energy balance, which include pancreas, small-intestinal epithelium, and plasma in C57BL/6Tac mice. Neonate mortality was also evaluated.

## Materials and methods

### Animals

Six-week old male and female C57BL/6Tac (Taconic, Oxnard, CA, USA) were grouped housed according to sex with free access to water and food and maintained on a 12-hour light/dark cycle (lights off at 1800 hours). Test diets composed of low-fat/no-sucrose standard lab rodent chow [(SD) Teklad 2020x Global Soy Protein-Free Extruded Rodent Diet; 16% kcal from fat, 60% kcals from carbohydrate, no sucrose], or Western diet [(WD) Research Diets D127098, New Brunswick, NJ, USA; 40% kcal from fat, 43% carbohydrates, mostly sucrose) (Table 1). All procedures met the U.S. National Institute of Health guidelines for care and use of laboratory animals, and were approved by the Institutional Animal Care and Use Committee (IACUC) of the University of California, Riverside.

**Table 1:** Relative energy content (% total kcal) of major nutrients in laboratory mouse diets.

Diet	Source	%Kcal Fat/ Source	%Kcal CHO/ Source	%Kcal Protein/ Source
**Soy-Free Standard Rodent Chow (SD)**	Teklad Diets	16%	60%	24%
	2020x	Soybean Oil	Mostly Starch	Wheat and Corn
**Western Diet (WD)**	Research Diets	40%	43%	17%
	D12079B	Milk Fat	Sucrose	Mostly Casein

### Breeding parameters

**Fig 1:**
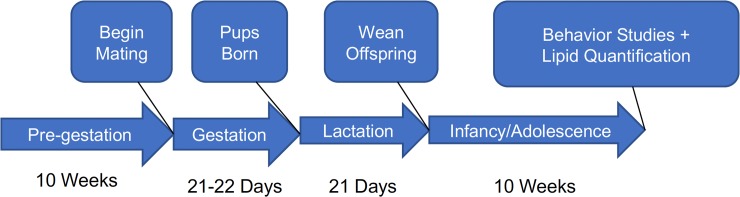
Experimental design. Female mice were fed a SD or WD for 10 weeks during the pregestation period, then males were introduced for mating. Females were then mainained on respective diets throughout the entirety of the experiment. Surviving offspring were weaned from dams at day 21 and placed on SD for 10 weeks of body mass monitoring. Behavioral analysis of feeding patterns were made after a 5 day acclimation period. At the conclusion of 10 weeks, tissues in offspring were collected and processed for analysis of endocannabinoid levels and expression of genes for components of the endocannabinoid system. Tissues from dams were collected during the pregestation phase and after 10 weeks on corresponding diet. Behavioral analysis in dams also occurred at this timepoint.

Female mice were fed either SD or WD during pre-gestation for 10 weeks, gestation, and lactation. Male mice were only given access to SD. Following 10 weeks, male mice were harem bred with female mice for mating at 1000 hr and separated at 1600 hr (see Fig 1 for experimental design). Food was removed during mating times and returned immediately after to avoid male consumption of WD diet. When a vaginal plug was observed during mating, females were single housed with their respective diets to give birth. Birth dates, litter sizes, neonate survival, and overall health of all animals were monitored daily. Body weights of offspring were recorded twice weekly. At postnatal day 21, pups were weaned, ear tagged, grouped housed with mice of same condition (i.e., those born from the same maternal test diet), and given access to only SD for the duration of the 10-week study. Pregnancies all occurred approximately at the same date. Thus, the outcome from all litters occurred at approximately the same date.

Notably, increased mortality of neonates was not an expected outcome or anticipated endpoint of our experimental design, which was aimed at evaluating feeding patterns and endocannabinoid system expression and function in peripheral organs of offspring born from dams maintained on WD or SD. Thus, despite daily monitoring of health and adherence to well-defined IACUC-approved humane endpoints for determining when animals will be removed from studies, treated, or euthanized, their implementation was not possible for mice in this study given that no signs of suffering or distress were observed for non-surviving neonates and that mortality of neonates occurred unexpectedly and exclusively within the first six days following birth. The cause of increased mortality is unknown but is associated with chronic maternal consumption of WD. Out of a total of 70 pups, 44 failed to survive by six days post-birth, at which time the remaining pups survived throughout the entirety of the study. All dams born from WD fed mothers suffered from at least one dead pup, with several damns suffering from complete mortality. Thus, litter sizes could not be adjusted to the same number of pups in the groups due to the variability of litter sizes and concomitant survival rates in litters born from WD fed mothers.

### Feeding behavior

Age-matched females (to those used in maternal studies) and surviving offspring were separated into single house feeding chambers (TSE Systems) and acclimated for 5 days prior to feeding behavior testing. Food and water intakes were obtained every 60 seconds. Baseline feeding behavior was monitored for 24 hours to assess daily intake patterns. In addition, a preference test between SD and WD was conducted after baseline reading. Animals had free access to either diet for 24 hours and their feeding behaviors were recorded. Feeding parameters included total caloric intake, average meal size, average rate of intake, average number of meals, average meal duration, average post-meal interval, and percentage of total meals between SD and WD (preference test).

### Tissue processing

#### Tissue collection

Isoflurane was used to anesthetize animals at time of tissue harvest (0900 to 1100 hours). Blood was collected by cardiac puncture and stored in EDTA-lined tubes on ice. Tubes were centrifuged (1500g for 10 minutes at 4°C) to obtain plasma. Pancreas was rapidly collected, washed with ice-chilled phosphate-buffer solution (PBS), and flash frozen in liquid nitrogen. Jejunum was rapidly collected, washed with ice-chilled PBS, sliced open longitudinally on a stainless steel plate kept on ice, scraped with a glass slide to separate mucosal layer, then mucosa was flash frozen in liquid nitrogen. All samples were stored at -80°C until time of processing.

#### Lipid extraction

Tissues were weighed and homogenized in 1.0 mL of methanol solution containing internal standards: [^2^H_5_]-2-arachidonoyl-*sn*-glycerol (2-AG), [^2^H_4_]-arachidonoylethanolamide (AEA), and [^2^H_4_]-oleoylethanolamide (OEA) (Cayman Chemical, Ann Arbor, MI, USA). Lipids were extracted using chloroform (2.0 mL) and washed with ultra-pure (0.2 μm filtered) water. Lipids were extracted from plasma using sterile 0.9% saline solution in lieu of water (0.1 mL plasma at expense of saline). Organic phases were collected and separated using silica gel column chromatography as previously described [40]. Eluate was gently dried under N_2_ stream (99.998% pure) and re-suspended in LCMS grade methanol:chloroform (9:1) solution [100 μL for plasma, 200 μL for tissue]. 1 μL was injected for analysis by ultra-performance liquid chromatography coupled with tandem mass spectrometry (UPLC-MS/MS) as described [40, 49].

#### Lipid analysis

Measurements of eCBs (2-AG and AEA) and related molecules [docosahexaenoylethanolamide (DHEA), docosahexaenoylglycerol (DHG)] were performed using methods previously described by our group [40, 49, 50]. Data was collected using an Acquity I Class UPLC system coupled to a Xevo TQ-S Mass Spectrometer (Waters, Milford, MA, USA) with accompanying electrospray ionization (ESI) interface. Lipids were separated on an Acquity UPLC BEH C18 column (2.1 × 50 mm i.d., 1.7 μm, Waters) with inline Acquity guard column (UPLC BEH C18 VanGuard Pre-column; 2.1 × 5 mm i.d., 1.7 μm, Waters), and eluted by a gradient of methanol in water (0.25% acetic acid, 5 mM ammonium acetate) according to the following gradient at a flow rate of 0.4 mL per min: 80% methanol 0.5 min, 80% to 100% methanol 0.5–2.5 min, 100% methanol 2.5–3 min, 100% - 80% methanol 3–3.1 min). Column temperature was maintained at 40°C, and samples were maintained in the sample manager at 10°C. Argon (99.998%) was used as collision gas. MS detection was in positive ion mode and capillary voltage set at 0.1 kV. Cone voltage and collision energy as follows, respectively:: 2-AG = 30v, 12v; AEA = 30v, 14v; OEA = 28v, 16v; DHEA = 30v, 16v; DHG = 34v, 14v; [^2^H_5_]-2-AG = 25v, 44v; [^2^H_4_]-AEA = 26v, 16v; [^2^H_4_]-OEA = 48v. 14v. Lipids were quantified using a stable isotope dilution method detecting protonated adducts of the molecular ions [M + H]+ in the multiple reaction monitoring (MRM) mode. Acyl migration from 2-AG to 1-AG is known to occur [51], thus all reported values for 2-AG represent the sum of 2-AG and 1-AG. Tissue processing and LCMS analysis from an individual experiment occurred independently of other experiments. Extracted ion chromatograms were used to quantify 2-AG (*m/z* = 379.2>287.26), AEA (*m/z* = 348.3>62.04), OEA (*m/z* = 326.3>62.08), DHEA (*m/z* = 372.3>91.02), DHG (*m/z* = 403.3>311.19), and [^2^H_5_] 2-AG (*m/z* = 384.2>93.4), [^2^H_4_] AEA (*m/z* = 352.3>66.11) and [^2^H_4_] OEA (*m/z* = 330.3>66.05), which were used as internal standards. One “blank” sample was processed as a control and analyzed in the same manner as all samples, except no tissue or plasma were included. This control revealed no detectable endocannabinoids and related molecules included in our analysis (see [49] for description of contaminants in standard glassware).

### Gene expression

Tissues were chosen at random to reflect a sample from each distinct litter in each of the two groups (SD vs. WD). Total RNA was extracted from jejunum mucosa and pancreas using QIAzol (Qiagen, Maryland, USA) and RNeasy (Qiagen, Germany) combined method, and generated first strand complementary DNA using M-MLV reverse transcriptase (Invitrogen, Carlsbad, CA). Surfaces for tissue collection and processing were sanitized using 70% EtOH solution followed by RNAse inhibitor (RNAse out, G-Biosciences, St. Louis, MO, USA) to maintain integrity of isolated RNA. Reverse transcription of RNA was performed with random hexamers (Invitrogen, Carlsbad, CA, USA) for 50 minutes at 37°C, RT-PCR was carried out using PrimePCR assays (BioRad, Irvine, CA, USA) with primers for cannabinoid receptor 1 and 2 (Cnr1, Cnr2), diacylglycerol α and β (DAGLα, DAGLβ), monoacylglycerol lipase (MGLL), N-acyl phosphatidylethanolamine-specific phospholipase D (NAPE-PLD), and fatty acid amide hydrolase (FAAH) using preconfigured SYBR green assay (BioRad, Irvine, CA, USA). Reactions were run in triplicate. Hprt was selected as a housekeeping gene for jejunum mucosa studies; β-actin was chosen as a housekeeping gene for pancreas studies. No changes in expression were found in conditions tested between respective housekeeping genes [Cq values for conditions, n = 5; hprt: SD born offspring, 24.51±0.84, WD born offspring, 24.47±0.77, not significant; β-actin: SD born offspring, 25.59±0.56, WD born offspring, 25.12±0.32, not significant].

### Statistical analyses

Data was analyzed using Graphpad Prism 7.0 software. Results are expressed as the mean ±S.E.M. Significant differences between groups were assessed using Student’s two-tailed t-test or two-way analysis of variance (ANOVA) with repeated or non-repeated measures with Sidak or Newman-Keuls post hoc analysis, respectively. Survival curves were analyzed using Mentel-Cox and Gehan-Breslow-Wilcoxon tests. Differences were considered significant if p<0.05. Grubbs’ tests for statistical outliers were performed on data from biochemical analyses. Statistical significance of any parameter was not affected by inclusion of outliers.

## Results

### Female mice fed Western diet display altered feeding behavior during pre-gestation phase

**Fig 2:**
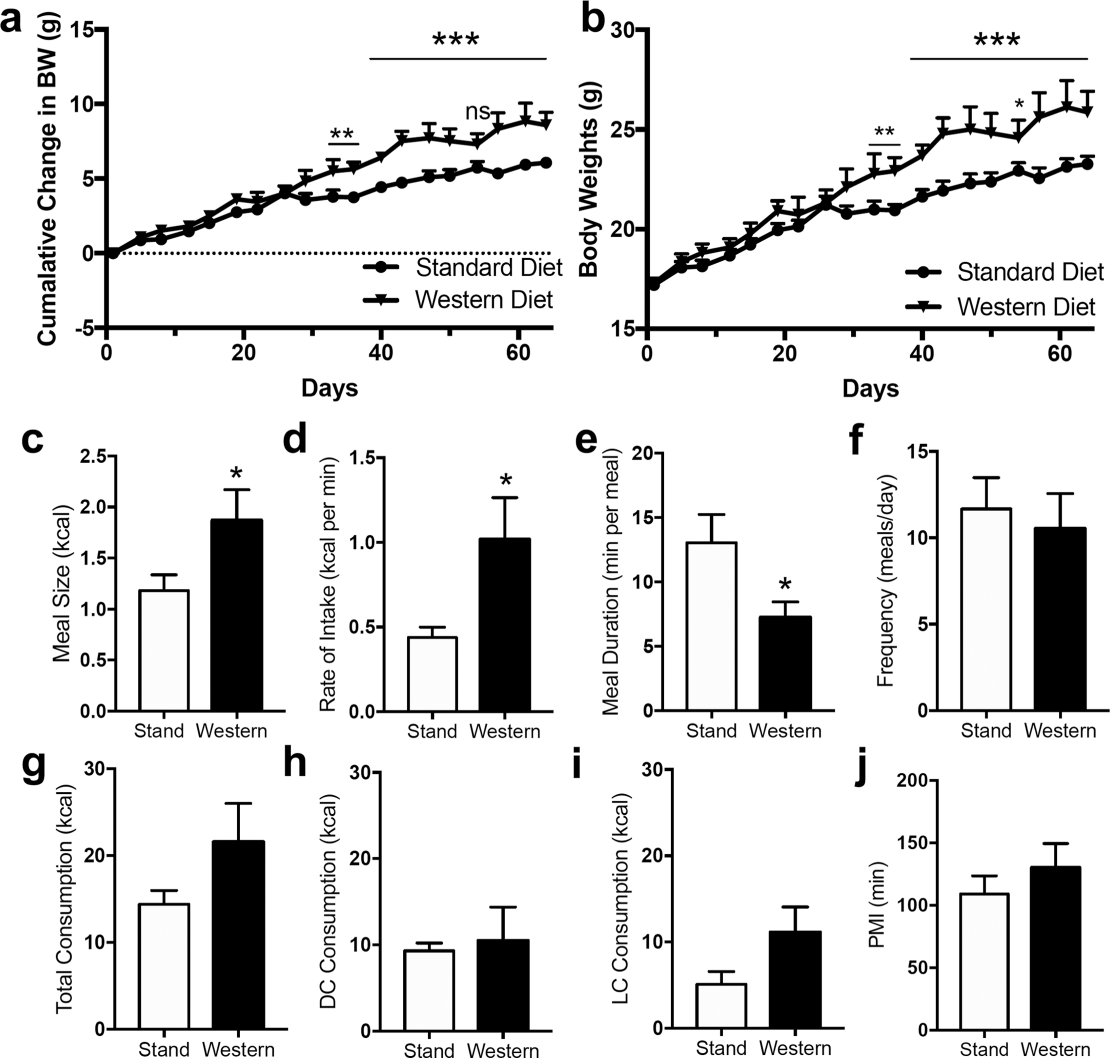
Chronic consumption of western diet results in altered feeding behavior in female mice. Female mice maintained for ten weeks (a, cumulative change in body weight; b, gross body weight) on a western diet (Western) become obese and display increases in 24 h meal size and rate of intake paired with a decrease in meal duration (c-e) when compared to mice maintained on a standard chow diet (Stand). Meal frequency, total caloric intake, dark cycle intake, light cycle intake, and post meal interval do not significantly differ between diets (f-j). Repeated measures two-way ANOVA, with Sidak's multiple comparison post hoc test, *** = p<0.001, ** = p<0.01, * = p<0.05 (a,b); unpaired Student's t-test (two-tailed), *** = p<0.001, * = p<0.05 between Stand and Western. Results are expressed as means ± SEM; n = 6 per condition.

Female mice fed Western diet (WD) ad-libitum over a time course of 10 weeks (age-matched to mice used in maternal experiments below), when compared to mice maintained on standard rodent chow (SD), gained weight at a higher rate based on change in body weight [Fig 2A, diet effect on cumulative change in body weight, *F*(1,5) = 18.89, p = 0.007; interaction between diet and time, *F*(18,90) = 4.182, p<0.0001)]; however, effect of diet on cumulative gross body was only significant when accounting for interaction between diet and time [Fig 2B, diet effect on cumulative gross body weight, *F*(1,5) = 5.3, p = 0.07; interaction between diet and time [*F*(18,90) = 4.182, p<0.0001)]. These effects were met with an increased average meal size (Fig 2C, *t* = 2.085, p = 0.039), increased rate of intake (Fig 2D, *t* = 2.382, p = 0.019), and lower meal duration (Fig 2E, *t* = 2.247, p = 0.026), over a 24-hour period. Other feeding parameters tested were not affected by diet, including meal frequency (Fig 2F, *t* = 0.424, p = 0.68), total caloric consumption (Fig 2G, *t* = 1.146, p = 0.279), dark cycle consumption (Fig 2H, t = 0.282, p = 0.781), light cycle consumption (Fig 2G, *t* = 1.179, p = 0.104), and post meal interval (Fig 2J, *t* = 0.864, p = 0.389). Total daily caloric consumption trended towards an increase but did not reach statistical significance (Fig 2G), which is in contrast to reports of significant increases in body weight and daily caloric intake in male mice fed a WD versus SD under similar conditions (see our [40]).

These results highlight important sex differences in feeding patterns in mice chronically maintained on WD and may represent differential expression of the eCB system and function among sexes in organs important for food intake and energy balance, including the upper small-intestinal epithelium [35, 52] and pancreas [21]. Indeed, when compared to male mice fed WD for 60 days that display increases in levels of the eCBs anandamide and 2-AG in the upper small intestinal epithelium and plasma [40], female mice fed WD displayed decreases and increases in levels of plasma 2-AG (Table 2, *t* = 2.654, p = 0.025) and anandamide (Table 2, *t* = 3.025, p = 0.0128) respectively, and no changes in levels of these eCBs in upper small intestinal epithelium when compared to mice fed SD (Table 2). Levels of the eCB-related molecule oleoylethanolamide (OEA) was increased in plasma of WD mice (Table 2, *t* = 3.223, p = 0.01), and docosahexaenoyl ethanolamide (DHEA) was decreased (Table 2, *t* = 3.605, p = 0.005) in upper small intestinal epithelium when compared to control mice fed SD.

**Table 2:** Lipid levels in blood plasma, jejunum mucosa, and pancreas.

	Tissue	Diet	2AG	AEA	OEA	DHEA	DHAG
**Mothers**	Plasma	SD	26.3 ± 3.09	1.73 ± 0.19	13.82 ± 0.56	1.717 ± 0.12	14.78 ± 2.70
		WD	*16.6 ± 1.95	*2.596 ± 0.21	*19.49 ± 1.52	1.658 ± 0.14	7.516 ± 3.72
	Jej. Mucosa	SD	46.03 ± 2.80	2.672 ± 0.35	47.49 ± 2.64	2.477 ± 0.32	9.401 ± 0.86
		WD	44.13 ± 2.34	2.415 ± 0.14	53.07 ± 2.81	**1.152 ± 0.18	*12.69 ± 0.70
	Pancreas	SD	11.07 ± 3.45	3.248 ± 0.24	105.1 ± 22.09	4.28 ± 0.52	2.236 ± 0.84
		WD	11.92 ± 5.31	5.275 ± 1.608	116.7 ± 29.00	3.185 ± 0.62	2.36 ± 0.98
**Offspring**	Plasma	SD	42.1 ± 4.94	1.007 ± 0.072	17.15 ± 1.45	1.412 ± 0.09	24.79 ± 0.81
**Males**		WD	42.68 ± 4.35	1.013 ± 0.06	15.72 ± 1.41	1.17 ± 0.09	*20.04 ± 1.46
	Jej. Mucosa	SD	49.35 ± 7.71	1.475 ± 0.28	101.7 ± 10.38	2.662 ± 0.26	9.41 ± 1.54
		WD	50.42 ± 7.34	1.696 ± 0.34	110.6 ± 16.35	2.821 ± 0.21	9.596 ± 1.53
	Pancreas	SD	35.41 ± 4.31	1.411 ± 0.27	27.82 ± 4.50	2.335 ± 0.41	4.744 ± 0.57
		WD	31.32 ± 3.03	1.115 ± 0.21	41.53 ± 11.83	2.468 ± 0.39	4.083 ± 0.34
**Offspring**	Plasma	SD	42.02 ± 4.74	0.9473 ± 0.05	18.11 ± 1.83	1.454 ± 0.07	10.61 ± 1.51
**Females**		WD	44.83 ± 4.37	0.6069 ± 0.18	13.34 ± 0.18	*1.151 ± 0.06	11.9 ± 1.69
	Jej. Mucosa	SD	76.74 ± 11.88	1.518 ± 0.33	117.9 ± 14.53	3.274 ± 0.41	9.911 ± 1.601
		WD	84.35 ± 6.34	1.547 ± 0.22	125 ± 16.62	3.599 ± 0.41	14.03 ± 1.58
	Pancreas	SD	68.69 ± 6.63	1.084 ± 0.26	28.73 ± 4.52	1.82 ± 0.30	5.659 ± 0.80
		WD	50.3 ± 10.17	1.189 ± 0.13	17.41 ± 1.49	2.075 ± 0.25	4.445 ± 0.90

Values +/- SEM for each analyte were compared across dietary conditions for all groups using Student’s two-tailed t-tests. Significant differences were denoted: ** = p<0.01, * = p<0.05, ns = p>0.05. Plasma = all analytes pmol per mL; jejunum mucosa = 2-AG and DHG nmol per mg tissue, and AEA, OEA, DHEA pmol per mg tissue; pancreas = 2-AG and DHG nmol per mg tissue, and AEA, OEA, DHEA pmol per mg tissue.

### Female mice fed WD display changes in endocannabinoid and related lipid profiles in plasma and jejunal epithelium during pre-gestation phase

Lipids were extracted from blood plasma, epithelium of the proximal small intestine (jejunum), and pancreas of female mice maintained on WD or SD for 10 weeks (age-matched to mice used in maternal experiments below). Female WD mice, when compared to those fed SD for 10 weeks, had significantly higher levels of plasma AEA (*t* = 3.025, p = 0.0128) and OEA (*t* = 3.223, p = 0.01), and reductions in 2-AG (*t* = 2.654, p = 0.024), but did not display differences in jejunal epithelium or pancreas (Table 2). Levels of the less characterized DHEA (*t* = 3.605, p = 0.005) and DHG (*t* = 2.963, p = 0.014) were also decreased and increased, respectively, in jejunal epithelium. These results are in contrast to male C57BL/6 mice similarly fed WD for 60 days, which were reported to have increases in plasma and jejunal epithelial 2-AG and AEA (see our [40]). This data highlights important sex differences in the impact of DIO on the peripheral eCB system, which may underlie differential feeding patterns (i.e., total caloric intake) in females (Fig 2) versus males (see our [40]).

### Offspring born from DIO mothers have lower rates of survival

**Fig 3:**
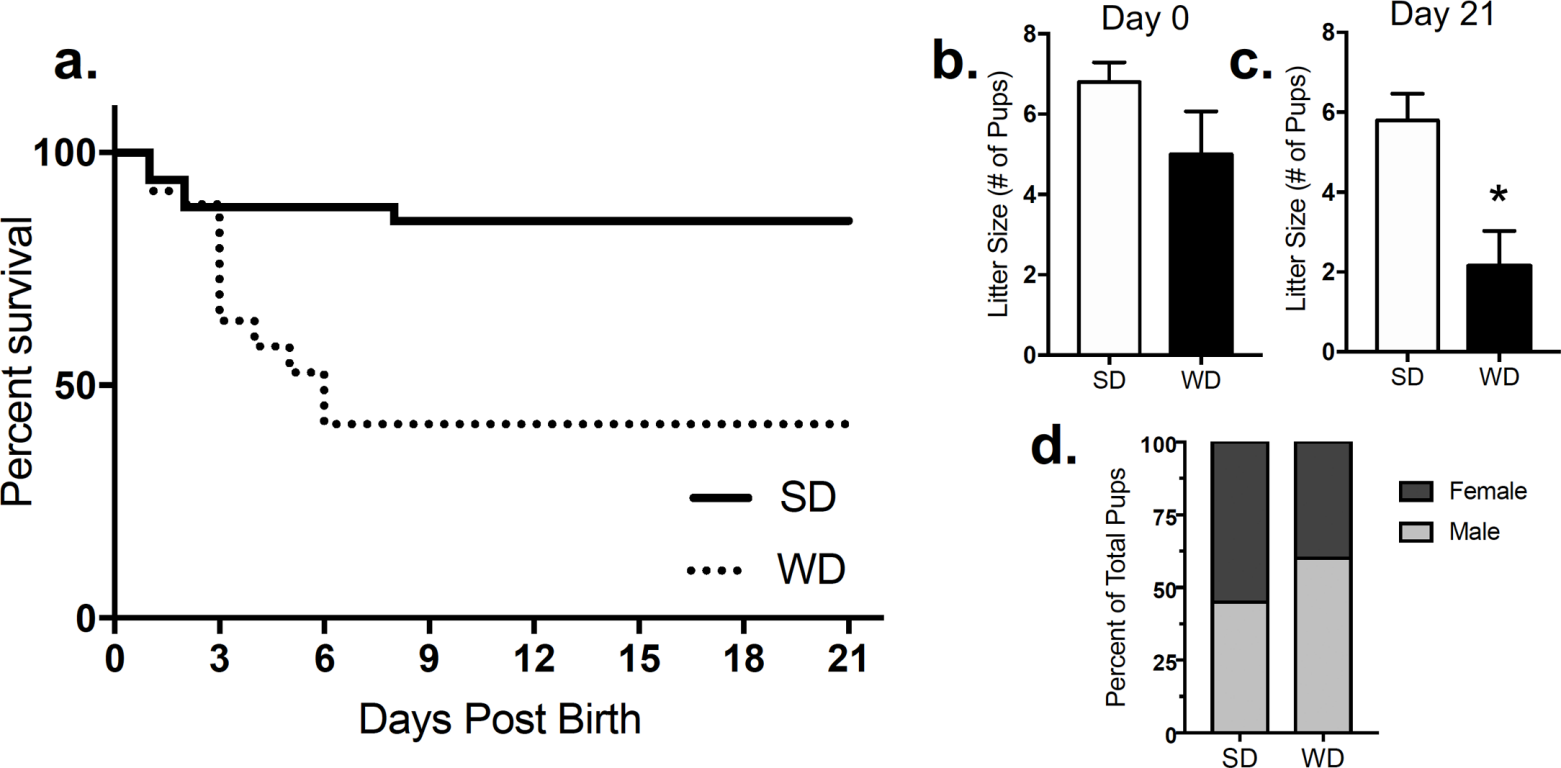
Chronic consumption of a maternal western diet increases mortality rate and male fraction in mice neonates. Mice born from mothers chronically fed a Western diet (WD) over 10 weeks have a lower survival rate when compared to mice born from dams fed a standard chow diet (SD) (a). The litter sizes between the two groups were similar in average number of pups at time of birth (b) but decreased in offspring born from DIO mothers at time of wean (c). An increase was found in the male fraction in groups born from WD fed mothers when compared to SD fed mothers (d, 60% vs. 45%). Survival data was analyzed using Mantel-Cox and Gehan-Breslow-Wilcoxon tests, HR 0.2309; 95% CI, 0.1032–0.5166, p = 0.0004. Litter size date was analyzed using unpaired Students t-tests (two-tailed): ns = p>0.05.

Female mice were fed a SD or WD during pre-gestation (10 weeks) and lactation phases (see Fig 1). Neonates born from DIO dams had lower rates of survival when compared to mice born to lean dams (Fig 3A, HR 0.2309; 95% CI, 0.1032–0.5166, p = 0.0004). All mortality occurred within the first six days following birth. Average litter sizes of the total offspring born between DIO and lean mothers did not differ at time of birth (Fig 3B, *t* = 0.691, p = 0.528). However, the average litter sizes of surviving offspring born from DIO mothers decreased by post-natal day 21 (Fig 3C, t = 3.06, p = 0.012)The remaining offspring that survived the lactation period born from DIO mice had a higher male fraction than surviving pups born from lean mothers (Fig 3D, 60% vs. 45%, respectively).

### Surviving offspring display similar feeding behaviors between groups

**Fig 4:**
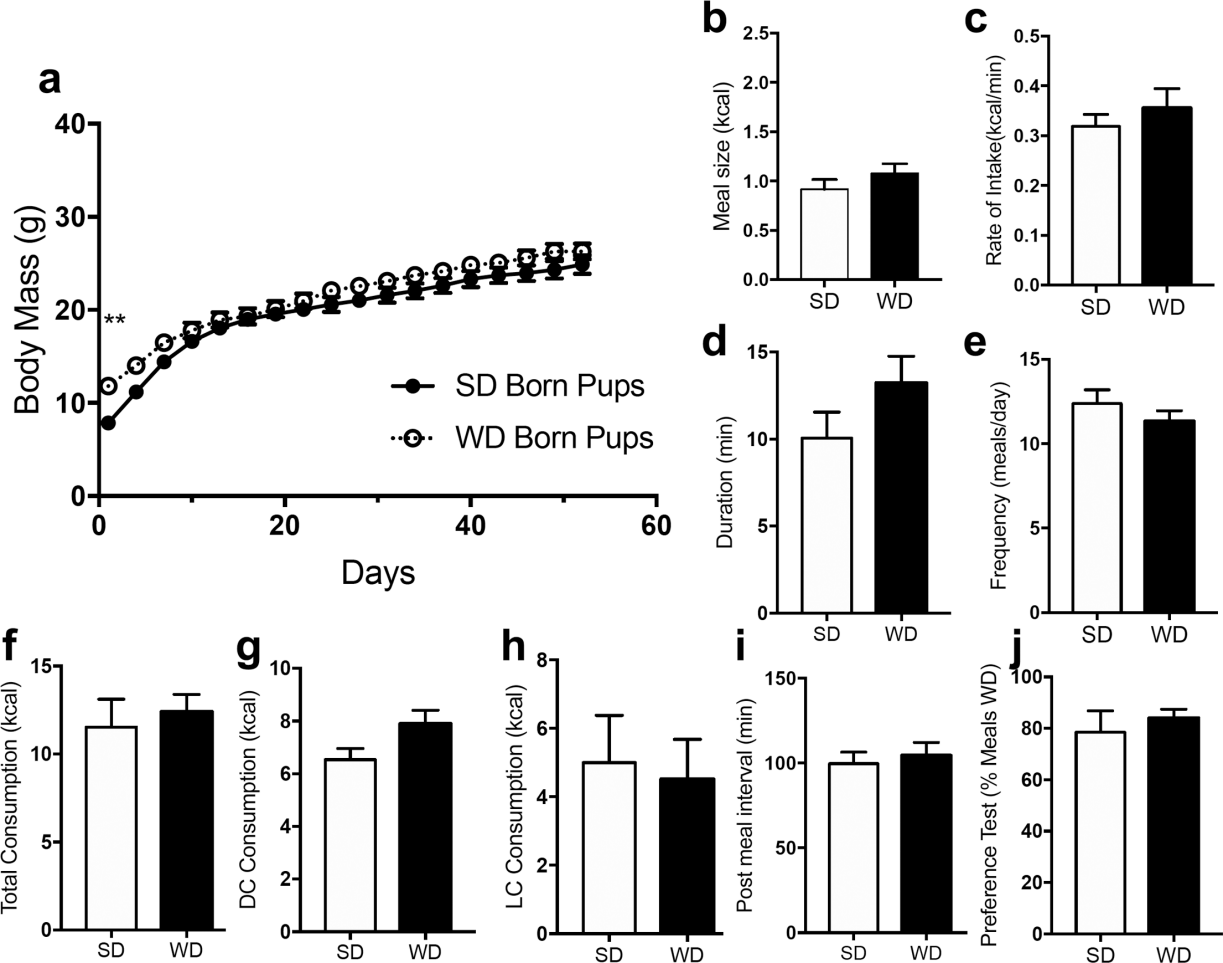
Surviving male mice born from DIO or lean dams do not display differences in feeding behavior. Male offspring weaned from dams fed a standard chow diet (SD) or a Western diet (WD) were given ad libitum access to standard chow for 8 weeks. Body weights were monitored and feeding behavior was tested. WD offspring had higher body mass at time of wean but body weights were rapidly normalized over the monitoring period (a). There were no differences meal size, rate of intake, meal duration, meal frequency, total caloric intake, dark cycle intake, light cycle intake, or in post meal interval (b-i). The offspring also displayed no difference in preference when given the choice between consuming a standard chow and highly palatable western diet (j). Data analyzed using repeated measures two-way ANOVA, with Sidak's multiple comparison post hoc test, ** = p<0.01, ns = p>0.05 (a); unpaired Student's t-test (two-tailed), ns = p>0.05 between SD and WD (b-j). Results are expressed as means ± SEM; n = 8/9 (SD/WD).

**Fig 5:**
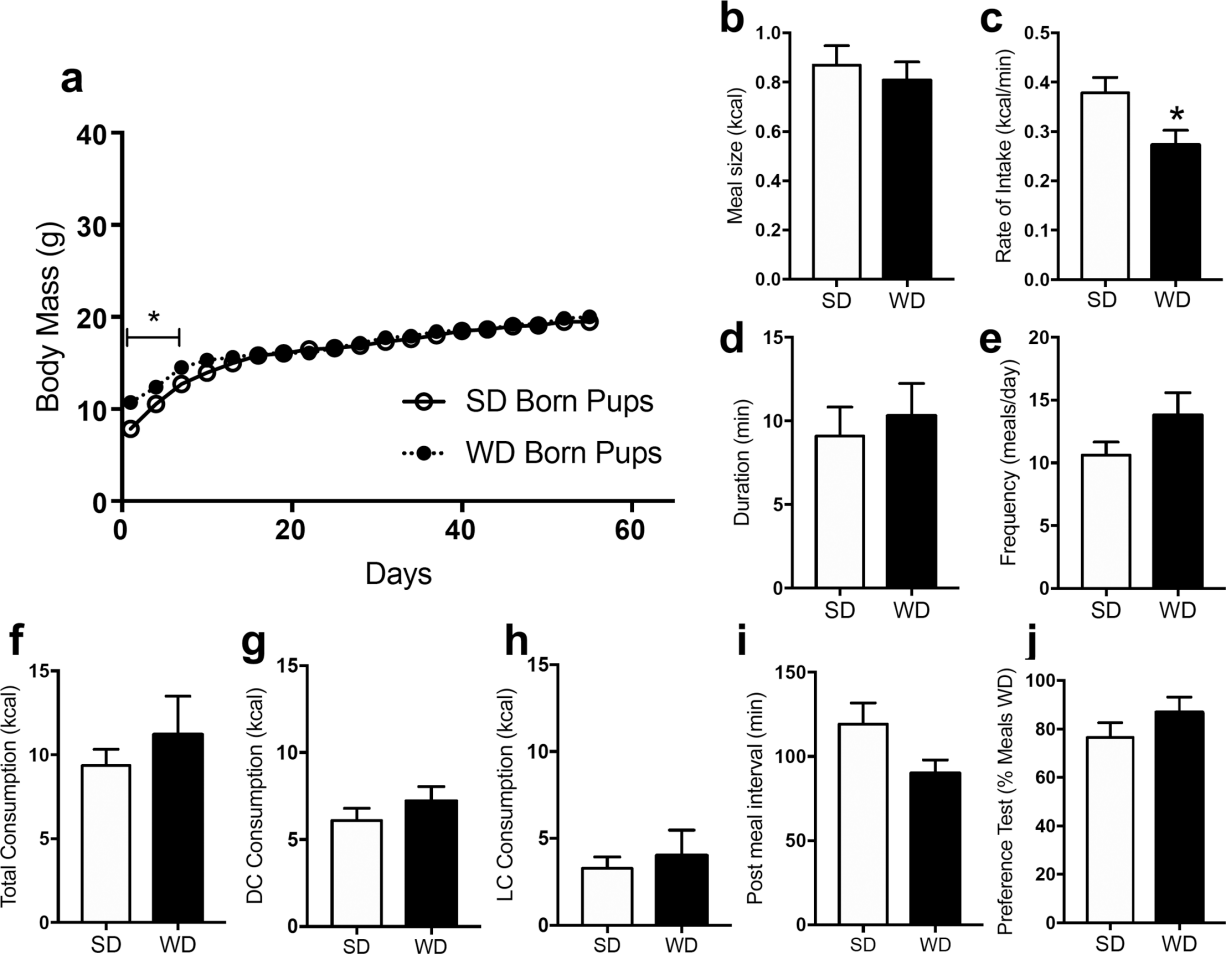
Surviving female mice born from DIO or lean dams display a small decrease in rate of intake. Female offspring weaned from dams fed a standard chow (SD) or a Western diet (WD) were given ad libitum access to standard chow for 8 weeks. Body weights were monitored and feeding behavior was tested. WD offspring had higher body mass at time of wean but body weights were rapidly normalized over the monitoring period (a). There were no differences meal size (a) but slight decrease in rate of intake between groups (b). Further, there were no differences in meal duration, meal frequency, total caloric intake, dark cycle intake, light cycle intake, or in post meal interval (d-i). The offspring also displayed no difference in preference when given the choice between consuming a standard chow and highly palatable western diet (j). Data analyzed using repeated measures two-way ANOVA, with Sidak's multiple comparison post hoc test, ** = p<0.01, ns = p>0.05 (a); unpaired Student's t-test (two-tailed), ns = p>0.05 between SD and WD (b-j). Results are expressed as means ± SEM; n = 8/4 (SD/WD).

Male (Fig 4A) and female (Fig 5A) offspring born from dams fed WD had higher body mass at time of wean when compared to those born from dams maintained on SD; however, body weights rapidly normalized over the monitoring period of WD mice to levels found in offspring born from SD dams. Male offspring did not display a difference in meal size (Fig 4B, *t* = 1.16, p = 0.244), rate of intake (Fig 4C, *t* = 0.798, p = 0.426), meal duration (Fig 4D, *t* = 0.146, p = 0.146), meal frequency (Fig 4E, *t* = 1.023, p = 0.323), total caloric intake (Fig 4F, *t* = 0.468, p = 0.647), dark cycle caloric intake (Fig 4G, *t* = 1.918, p = 0.066), light cycle caloric intake (Fig 4H, *t* = 0.276, p = 0.787), post meal interval (Fig 4I, *t* = 0.449, p = 0.654), or preference between WD and SD (Fig 4J, *t* = 0.618, 0.546). Female offspring did not display a difference in meal size (Fig 5B, *t* = 0.542, p = 0.589), meal duration (Fig 5D, *t* = 0.446, p = 0.656), meal frequency (Fig 5E, *t* = 1.609, p = 0.139), total caloric intake (Fig 5F, *t* = 0.883, p = 0.398), dark cycle caloric intake (Fig 5G, *t* = 0.938), light cycle caloric intake (Fig 5H, *t* = 0.543, p = 0.599), post meal interval (Fig 5I, *t* = 1.753, p = 0.082), or preference between WD and SD (Fig 5J, *t* = 1.048, p = 0.3191). Female offspring born from WD dams had a slightly decreased rate of intake (Fig 5C, *t* = 2.304, p = 0.023) when compared to female offspring born from SD controls.

### Surviving offspring display similar endocannabinoid and related lipid profiles irrespective of dam’s diet

Lipids were extracted from blood plasma, epithelium of the proximal small intestine (jejunum), and pancreas from male and female offspring from dams maintained on WD or SD for 10 weeks prior to mating and through to weaning (Table 2). Irrespective of dams’ diet, no changes were found in levels of 2-AG, AEA, or OEA in plasma, jejunal epithelium, or pancreas; however, plasma levels of DHG (*t* = 2.727, p = 0.017) in male offspring born from WD dams, and DHEA (*t* = 2.955, p = 0.018) in female offspring born from WD dams, were moderately decreased.

### Male offspring display changes in expression of mRNA for select endocannabinoid system components in jejunal epithelium

**Fig 6:**
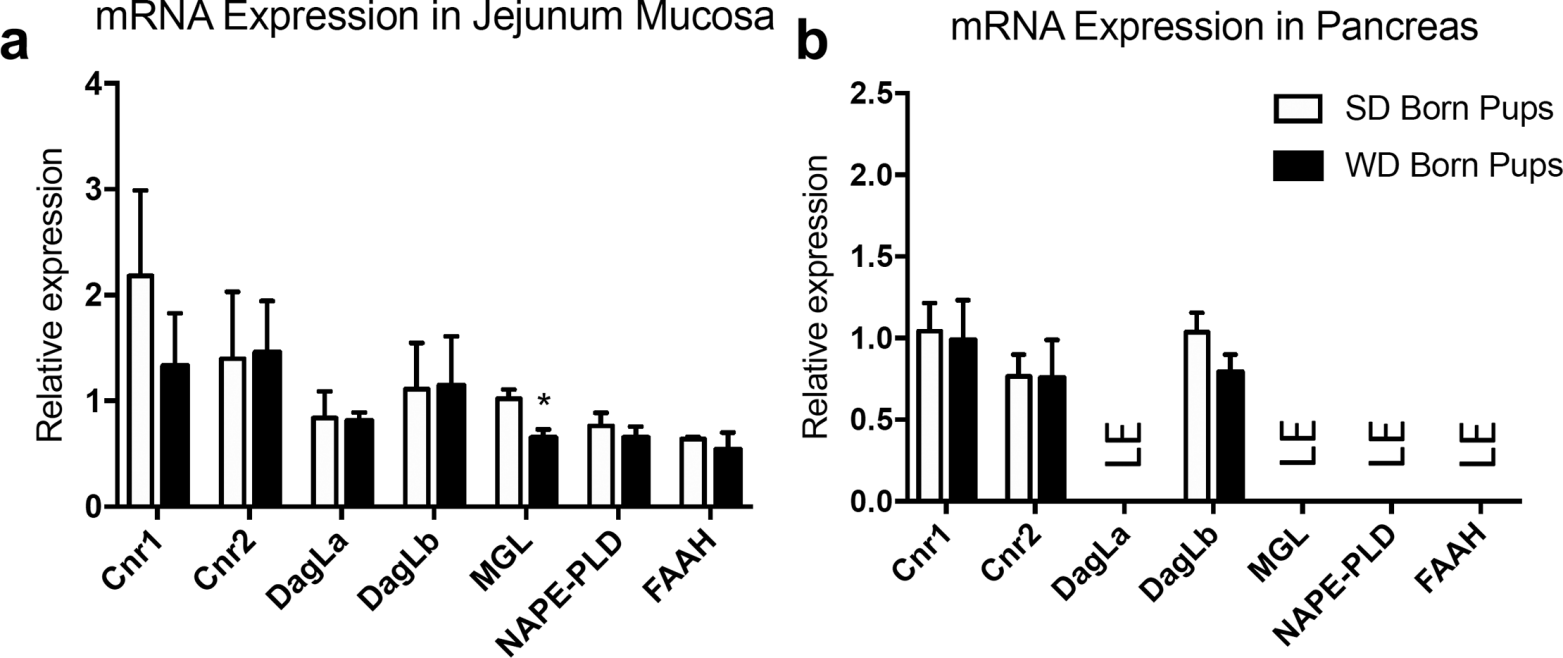
Male mice born from DIO dams display decreases in monoacylglycerol lipase (MGL) expression in jejunum mucosa. Expression of mRNA encoding MGL was significantly lower the in upper small intestinal epithelium of offspring born from WD dams versus SD dams. No changes were found between groups in expression of CB1R (Cnr1), CB2R (Cnr2), diacylglycerol lipase α/β (DagLa, DagLb), n-acyl phosphatidylethanolamine phospholipase-D (NAPE-PLD) and fatty acid amide hydrolase (FAAH) did not differ between tested groups in jejunum mucosa (a). In pancreas, Cnr1, Cnr2, and DagLb showed no differences between tested groups, while mRNA expression of DagLa, MGL, NAPE-PLD, and FAAH not detected (b). Data was analyzed using multiple student t tests. * = p<0.05, ns = not significant. Results are expressed as means ± SEM; n = 3–5 per condition in triplicate. LE = limited expression.

**Fig 7:**
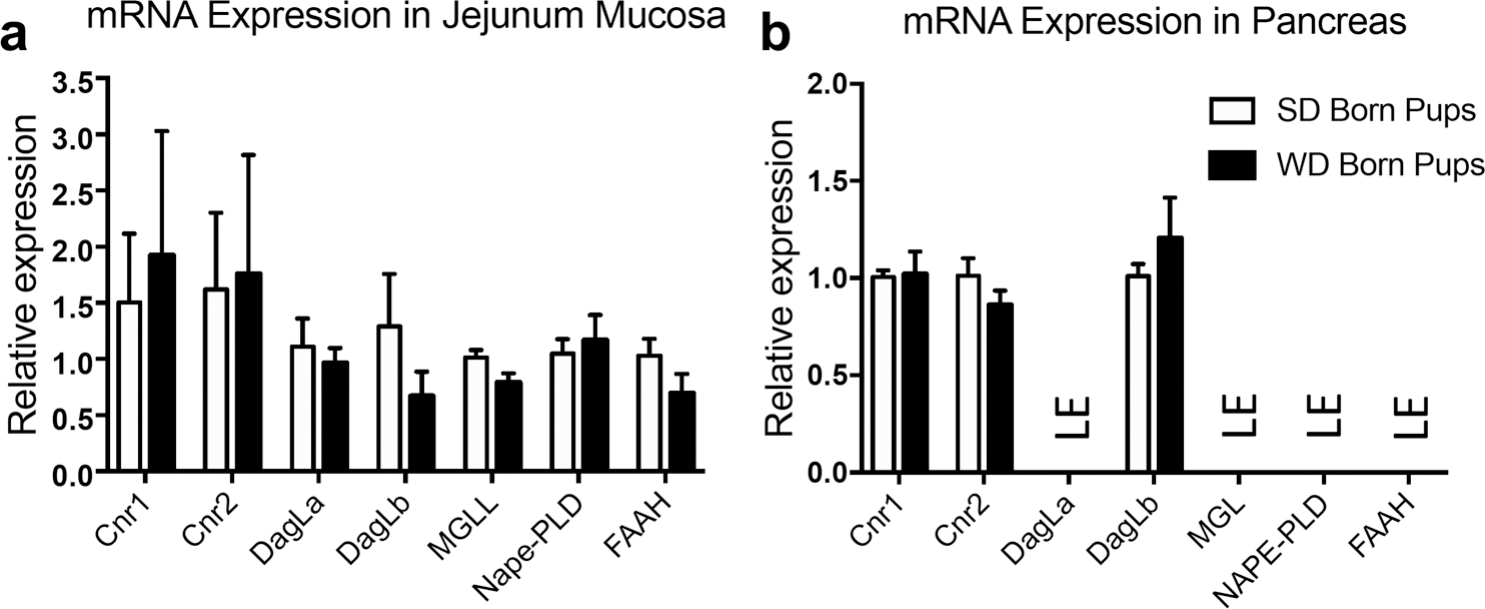
Female mice born from dams maintained on WD or SD display similar mRNA expression of key endocannabinoid system genes. Expression of mRNA encoding CB1R (Cnr1), CB2R (Cnr2), diacylglycerol lipase α/β (DagLa, DagLb), monoacylglycerol lipase (MGL), n-acyl phosphatidylethanolamine phospholipase-D (NAPE-PLD) and fatty acid amide hydrolase (FAAH) showed no changes between tested groups in jejunum mucosa (a). In pancreas, Cnr1, Cnr2, and DagLb showed no differences between tested groups while mRNA expression of DagLa, MGL, NAPE-PLD, and FAAH were unable to be quantified with the techniques described (b). Data was analyzed using multiple student t tests. ns = not significant. Results are expressed as means ± SEM; n = 4 per condition in triplicate. LE = limited expression.

We next evaluated expression of mRNA for components of the eCB system in jejunal epithelium and pancreas from male (Fig 6) and female (Fig 7) offspring from dams maintained on WD or SD for 10 weeks prior to mating and through to weaning. Expression of mRNA for the monoacylglyerol (e.g., 2-AG, DHG) degradative enzyme, monoacylglycerol lipase (MGL), was decreased in jejunal epithelium of male mice born from WD dams (Fig 6A), when compared to those born from SD dam, (*t* = 3.1, p = 0.017). In contrast, no changes in mRNA expression were found in the same tissue for CB_1_Rs (Cnr1; *t* = 0.897, p = 0.396) and CB_2_Rs (Cnr2; *t* = 0.069, p = 0.947), and the monoacylglycerol (e.g., 2-AG, DHG) biosynthetic enzymes, diacylglycerol lipase alpha (DagLa; *t* = 0.147, p = 0.888) and diacylglycerol lipase beta (DagLb; *t* = 0.491, p = 0.962), as well as no changes in expression of mRNA for the fatty acid ethanolamide (e.g., AEA, OEA, DHEA) biosynthetic enzyme, NAPE-PLD (*t* = 0.725, p = 0.492), or degradative enzyme, fatty acid amide hydrolase (FAAH; *t* = 0.475, p = 0.651). In male pancreas (Fig 6B), there were no changes in Cnr1 (*t* = 0.181, p = 0.861), Cnr2 (*t* = 0.042, p = 0.967), or DagLb (*t* = 1.516, p = 0.173) between groups born from mothers maintained on WD or SD. DagLa, MGL, NAPE-PLD, and FAAH were unable to be quantified due to low expression (LE). In female jejunal epithelium, (Fig 7A), no changes were found in Cnr1 (*t* = 0.331, p = 0.752), Cnr2 (*t* = 0.106, p = 0.919), DagLa (*t* = 0.513, p = 0.626), DagLb (*t* = 1.209, p = 0.272), MGL (*t* = 2.074, p = 0.083), NAPE-PLD (*t* = 0.449, p = 0.669), or FAAH (*t* = 0.192, p = 1.47). In female pancreas (Fig 7B), there were no changes in expression of Cnr1 (*t* = 0.921, p = 0.103), Cnr2 (*t* = 1.302, p = 0.241), or DagLb (*t* = 892, p = 0.407). DagLa, MGL, NAPE-PLD, and FAAH were unable to be quantified due to low expression (LE).

## Discussion

These studies suggest that maternal diet is an important predictor of survival in neonate C57BL/6Tac mice, and maternal WD is not associated with disruptions in feeding patterns or marked abnormalities in eCB system expression or function in jejunal epithelium, pancreas, or plasma in surviving offspring maintained on a SD for 10 weeks.

DIO is associated with a host of metabolic abnormalities that include type-2 diabetes [5, 6]. Obesity rates have increased over the past several decades in human females of child-bearing age, and obesity during gestation is associated with abnormal metabolic profiles in offspring, which may include an epigenetic component in these outcomes [53]. Thus, research aimed at exploring the impact of maternal diet on feeding behavior and glucose homeostasis is critical. In the present study, chronic exposure to WD greatly influenced offspring survival within the first 6 days following birth. Female mice were maintained on a WD for 10 weeks, at which time they displayed significantly increased body weight gain and altered feeding behaviors. Maternal obesity led to high rates of neonate mortality, with a higher male fraction in surviving pups. Our results are consistent with reports of increased mortality in rat pups born from mothers that were exposed to a highly-palatable “cafeteria diet” (i.e., chocolate candy bars) for eight weeks prior to mating and through to weaning [54]. Importantly, however, we found a much higher rate of mortality in our studies (above 50% mortality by 6 days post-birth in pups born from WD dams) when compared to studies by Ramirez-Lopez and colleagues (4.3% pups died at birth and about 10% during lactation by 21 days) [54]. It is plausible that dietary composition plays a large role in these effects and WD in our studies, which is high in milk fat (40% total diet kcals) and sucrose (29% total diet kcals), leads to considerably higher neonate deaths. A comprehensive analysis of the impact of specific dietary components (i.e., various types of fats and carbohydrates) on neonate survival in rodents remains.

Surviving offspring were then monitored for 10 weeks and feeding behaviors were assessed. Surviving offspring did not display notable differences in body weights or feeding parameters at 10 weeks of age (i.e., total caloric intake, meal size, meal duration, meal frequency, rate of intake, or preference tests). Other groups report increased body weights and food intake in mice or rats born from dams maintained on high-fat diets versus standard diets, but these effects were mostly apparent after 8–10 weeks post-weaning [13, 17, 55]. Thus, changes in feeding behaviors and body weight change under our conditions may occur at later timepoints from those included in our analysis (i.e., 10 weeks post-weaning). Furthermore, in contrast to our findings in mice that offspring born from dams maintained on WD or SD displayed no differences in preference for WD during a 24-h test, rats born from dams maintained on a “cafeteria diet” were reported to display “an exacerbated preference for fatty, sugary and salty foods at the expense of protein-rich foods” [56]. These results may reflect important species differences and differential impact of specific nutrients on palatable food preference. Future studies should include tracking the impact of maternal DIO on feeding patterns of offspring born from WD or SD dams over extended periods of time (i.e., greater than 10 weeks).

We next analyzed levels of eCBs (i.e., the fatty acid ethanolamide, AEA, and the monoacylglycerol, 2-AG) and related fatty acid ethanolamides (i.e., OEA and DHEA) and monoacylglycerols (i.e., DHG) in plasma, upper small intestinal epithelium, and pancreas. We found no changes in the eCBs in organs tested between groups born from dams chronically fed WD or SD, and modest reductions in levels of DHG in male plasma and DHEA in female plasma of those born from WD dams. DHG and DHEA are synthesized from the omega-3 fatty acid, docosahexaenoic acid (DHA, 22:6n-3). Their physiological relevance, however, is not well-established but may include roles in inflammation and neural development [57, 58]. It is plausible that lower levels of these molecules in plasma may play a role in systemic inflammation associated with DIO; however, a direct test of this hypothesis remains. Furthermore, we found no appreciable changes in expression of eCB biosynthetic and degradative enzymes in small intestinal epithelium and pancreas of males and females, with the exception of a small but significant reduction in expression of the 2-AG degradative enzyme, MGL, in males. The latter effect suggests a possible remodeling of the eCB metabolic machinery in the small intestinal epithelium in male mice born from mothers maintained on WD; however, no changes in levels of select monoacylglycerols (i.e., 2-AG and DHG) were found, which raises the question of the physiological significance of this change in expression of MGL. A complete analysis of the expression in tissue of a wider variety of monoacylglycerols under our conditions is warranted.

Notably, we analyzed eCB expression in organs of pups 10 weeks after weaning and maintenance on SD. Thus, we cannot rule out that changes in expression or function of the eCB system in pancreas and small intestinal epithelium occur in pups born from WD mothers at earlier time points and is transient, or at later time points from our analysis of eCB system expression and feeding behavior in pups (i.e., 10 weeks after weaning). Indeed, other groups reported sex-specific changes in eCB system expression in white and brown adipose depots in rat pups born from mothers maintained on a high-fat diet versus standard; however, in contrast to our experiments, their analysis occurred immediately after weaning [15]. Similarly, Ramirez-Lopez and colleagues reported reductions at birth in levels of the eCBs, AEA and 2-AG, in the hypothalamus of male rats born from mothers maintained on a high-calorie test diet versus standard chow during the pregestational and gestational periods [54]. Furthermore, rats born to mothers maintained on either a palatable chocolate diet in combination with standard chow (ad-libitum access to both) or control rats given access to only standard chow for 8 weeks displayed very small decreases in body weight gain over a period of nineteen weeks [16]. The authors then analyzed expression of eCB system components in brain, liver, and adipose tissue in these rats at nineteen weeks postnatal and found a variety of sex-specific changes in expression of mRNA for several components of the eCB system (see for details [16]). Collectively, these studies reveal possible changes in eCB system function or expression in several organs immediately after birth [54] or weaning [15], and at much later timepoints (i.e., nineteen weeks postnatal [16]) in offspring born from dams consuming high-energy diets. Furthermore, under our conditions, similar expression and function of the eCB system between groups in the upper small intestinal epithelium–particularly given its critical role in feeding behavior [45–47, 49]–may underlie a lack of change in feeding patterns or palatable food preferences observed between mice born from WD or SD dams at the time point analyzed in our experiments.

## Conclusions

Our studies reveal large increases in mortality in neonates born from dams maintained on a WD for 10 weeks before mating, during gestation, and through to weaning of pups. Mortality was restricted to the first six days after birth. Furthermore, changes in eCB system function and expression in these offspring, when compared to dams maintained on a control SD, were largely absent at 10 weeks post-weaning in small-intestinal epithelium, pancreas, and plasma. Future studies under our conditions should include a comprehensive temporal evaluation of eCB system expression and function in small intestinal epithelium, pancreas, and plasma of pups born from mothers maintained on WD and SD, from immediately after weaning through to time points after 10 weeks post-weaning. Furthermore, despite a lack of marked changes in eCB system profile or feeding behavior in offspring born from dams maintained on WD, it is important to consider that behavioral and biochemical analysis of the animals tested under our conditions were performed on surviving pups, which may be considered “extraordinary”. It is possible that the neonates that died within six days following their birth had significant changes in eCB system and other regulatory factors affecting feeding and glucose homeostasis that led to their failure to thrive. A test of this hypothesis, however, remains, but is difficult given the inability to predict when mice will die, and which mice will survive.

## Supporting information

S1 File Raw data file. (XLSX)
